# How Does Patient Safety Culture in the Surgical Departments Compare to the Rest of the County Hospitals in Xiaogan City of China?

**DOI:** 10.3390/ijerph14101123

**Published:** 2017-09-26

**Authors:** Manli Wang, Hongbing Tao

**Affiliations:** Department of Health Management, School of Medicine and Health Management, Tongji Medical College, Huazhong University of Science and Technology, No. 13 Hangkong Road, Wuhan 430030, China; wangmanli1237@hust.edu.cn

**Keywords:** patient safety culture, HSOPSC, surgical departments, county hospitals

## Abstract

*Objectives*: Patient safety culture affects patient safety and the performance of hospitals. The Hospital Survey on Patient Safety Culture (HSOPSC) is generally used to assess the safety culture in hospitals and unit levels. However, only a few studies in China have measured surgical settings compared with other units in county hospitals using the HSOPSC. This study aims to assess the strengths and weaknesses of surgical departments compared with all other departments in county hospitals in China with HSOPSC. *Design*: This research is a cross-sectional study. *Methods*: In 2015, a Chinese translation of HSOPSC was administered to 1379 staff from sampled departments from 19 county hospitals in Xiaogan City (Hubei Province, China) using a simple random and cluster sampling method. *Outcome Measures*: The HSOPSC was completed by 1379 participants. The percent positive ratings (PPRs) of 12 dimensions (i.e., teamwork within units, organizational learning and continuous improvement, staffing, non-punitive response to errors, supervisor/ manager expectations and actions promoting patient safety, feedback and communication about errors, communication openness, hospital handoffs and transitions, teamwork across hospital units, hospital management support for patient safety, overall perception of safety, as well as frequency of events reported) and the positive proportion of outcome variables (patient safety grade and number of events reported) between surgical departments and other departments were compared with *t*-tests and X^2^ tests, respectively. A multiple regression analysis was conducted, with the outcome dimensions serving as dependent variables and basic characteristics and other dimensions serving as independent variables. Similarly, ordinal logistic regression was used to explore the influencing factors of two categorical outcomes. *Results*: A total of 56.49% of respondents were from surgical departments. The PPRs for “teamwork within units” and “organizational learning and continuous improvement” were ≥75%, which denoted strengths, and the PPRs for “staffing” and “non-punitive response to errors” were ≤50%, which denoted weaknesses in surgical units and other units. Three dimensions for surgical departments were weaker than those for other departments (*p* < 0.05). The staff from surgical units reported more events compared with the other units, but only a few respondents in surgical settings evaluated patient safety grade as good/excellent. Four dimensions influenced patient safety grade, and three dimensions influenced event reporting in surgical units. *Conclusions*: Strategies including recruiting workers, using the reporting system, and building a non-punitive culture should be adopted in the surgical units of county hospitals in China to improve safety culture. Supervisors should also prioritise patient safety.

## 1. Introduction

Patient safety is considered to be crucial to health-care quality and is one of the major parameters monitored by all health-care organizations around the world [1]. In 1993, the UK Health and Safety Commission defined safety culture as the “product of the individual and group values, attitudes, competencies and patterns of behavior that determine the commitment to, and the style and proficiency of, an organization’s health and safety program” [2]. In 1998, Lucien, a pediatric surgeon and pioneer in the patient safety movement, described the prevailing safety culture as that of “anger, blame, frustration and distrust regarding health care errors” [3]. Since the release of the Institute of Medicine report “To Err is Human” [4], patient safety has received extensively increasing attention in many countries, including China. A positive safety culture improves hospitals’ patient safety performance, whereas a negative safety culture encourages hospitals to address issues in patient safety management [3]. Pronovost et al. asserted that assessing patient safety culture is the first step to improve safe care in hospitals [5]. Over the past decade, researchers have developed several instruments to evaluate patient safety culture [6,7,8]. Despite the largely independent development, these instruments feature similar psychometric properties and measure similar dimensions [9]. All these instruments use Likert scales, mostly measure attitudes of individuals, and nearly all cover five dimensions of patient safety climate, namely, leadership, policies as well as procedures, staffing, communication, and reporting [10]. One of these measurement tools is the Hospital Survey on Patient Safety Culture (HSOPSC), which was first introduced by the Agency of Healthcare Research and Quality (AHRQ) in America. The HSOPSC has been widely used because of its good reliability (Cronbach coefficient = 0.63–0.84) as well as validity (R = 0.23–0.60), detailed measurement structure, and unified quantitative standard [11].

An increasing number of studies have assessed patient safety culture, thereby revealing differences in patient safety culture across professions [12,13], units [14], hospitals [13,15], and countries [16]. In certain studies, unit-level assessment has emerged as a common topic. Many researchers have measured patient safety culture in intensive care units (ICUs) in their countries [17]. For example, Kho et al. [18] corroborated that three major safety themes require solutions: appropriate staffing, medication safety, and the improvement of the bedside care of obese patients. Other units’ patient safety culture [19,20,21] was also assessed by many scholars with different measurement tools. Liu [22] conducted a case study in an outpatient setting in China to measure its patient safety culture and affirmed that strong “teamwork within units”, a common area of strength, could fuel the concealment of errors. Hoffmann [23] carried out an open randomized controlled trial and evaluated the effects on patient safety culture in general practice. Other surveys assessed surgery and general practice units [20].

Surgical departments, including operating rooms, anesthesia units, obstetrics and gynecology departments, and other surgical units, are high hazard settings with a high potential for patient harm. The safety culture in these departments has been measured in certain studies. Bognár et al. [24] evaluated safety climate in a pediatric cardiac surgery unit, but they did not compare surgery units with the rest of the hospital units. Kaafarani et al. [25] assessed the patient safety culture in operating rooms and post-anesthesia units, but this survey was conducted with the Patient Safety Climate in Healthcare Organizations. Several studies measured surgical settings, particularly in China, using the HSOPSC. Our previous study [26] evaluated the patient safety culture in a surgical unit relative to that in other units on the basis of the HSOPSC, but we conducted the study in only one tertiary hospital. The research into safety culture in surgical units and operating rooms provides a reference for other departments. On the basis of previous studies, our research will continually reveal differences in patient safety culture across different departments in county hospitals.

Surgical departments in county hospitals consist of people with varying levels of expertise and highly advanced equipment, vulnerable patients, and limited time to treat patients [27]. In addition, surgical departments are complex and have high potential hazard for patient harm and adverse events [28]. More than half of all adverse events in hospitals (51–62%) occur in surgical settings [6,29]. Promoting highly reliable care in operating environments requires a strong patient safety culture [3]. Consequently, safety culture assessment in surgical departments is an urgent concern. 

Xiaogan City is located in northeast Hubei Province, China. According to the Xiaogan Statistical Information Network, in 2014, Xiaogan City had an area of 8910 km^2^, and its resident population was 4.8661 million, and the regional gross domestic product (GDP) was 135.472 billion Renminbi (RMB). In 2014, Xiaogan City had a total of 423 health institutions (excluding village clinics), of which 45 were hospitals. It is one of the members of the Wuhan city circle and one of the most potential and competitive cities in the central region of China. The aim of our study was to examine the strengths and weaknesses of surgical departments compared with those of other departments in county hospitals in Xiaogan City of Hubei Province, China as well as to find out the factors affecting patient safety culture in hospitals at the county level. The results are expected to provide a reference for the policy makers and managers of county hospitals as they seek to improve patient safety culture in surgical settings by suggesting targeted recommendations for hospital unit levels on the basis of the HSOPSC.

## 2. Methods

### 2.1. Design

This study employed a cross-sectional survey.

### 2.2. Setting and Sampling

All 19 county hospitals in Xiaogan City located in northeast Hubei Province, China, were taken as the sample hospitals in this study. In 2015, the average in-service medical staff in these 19 county hospitals was 428, and the average open beds were 329. In the same year, the average diagnosis and treatment visits in the 19 hospitals were 190,543, of which 14,440 were outpatients. The average annual income of these 19 county hospitals was 108.28 million RMB in 2014. Subsequently, in every county hospital, the surgical departments (including the departments of general surgery, orthopedics, obstetrics and gynecology, emergency rooms, ICUs, urology, neurosurgery, and other comprehensive surgical units) and other hospital departments (referring to settings of most internal medicine units, traditional Chinese medicine, rehabilitation, nursing, B ultrasonic room, medical department, and other units) were selected with a simple random sampling method. All the doctors and nurses in the selected surgical departments and other departments were investigated using the HSOPSC with a cluster sampling method. Finally, 1379 questionnaires were collected, and all these 1379 questionnaires were deemed valid.

### 2.3. Instrument

The Chinese version of the HSOPSC was previously validated for paper distribution in our previous study [26]. The HSOPSC comprised 42 items, and the participants responded to the items on a five-point Likert scale ranging from “strongly disagree” to “strongly agree” or from “never” to “always”. The result variable “patient safety grade” was scored from 1 to 4 as follows: (1) excellent, (2) good, (3) acceptable, and (4) poor/failing. The result variable “number of events reported” by the respondents during the last 12 months was scored from 1 to 5: (1) “no events”, (2) “1 to 2 events”, (3) “3 to 5 events”, (4) “6 to 10 events”, and (5) “>10 events”. These two result variables are not part of the 42 items in the questionnaire, but were asked to be scored by the respondents separately.

The HSOPSC has well-established psychometric properties, including factor analysis, reliability, and item analysis [9,30]. To ensure that the concepts were correctly worded and conceptualized, we pilot-tested the readability and functionality of this questionnaire on several health-care workers and research personnel. After the pilot testing, factor analysis was conducted to measure the validity of the questionnaire in our previous study [26]. In the factor analysis, all 42 items were retained and divided into 12 dimensions, each of which included three to four items. The characteristics of the respondents were also included. The authors determined that the order of the questionnaire was not suitable for the habits of the Chinese. Accordingly, background information was moved to the beginning, and the questions on “patient safety grade” and “number of events reported” were moved toward the end of the questionnaire.

### 2.4. Data Collection

The study was carried out in August 2015. After receiving training in the hospital research department about the questionnaire’s content, the consultants went to the sample units from the 19 hospitals to distribute the questionnaires to the medical personnel. The timeline was set to 1 week, and the medical personnel received oral and e-mail reminders from the consultants every day until they completed the questionnaires. Some administrative staff and logistics staff didn’t participate in our survey because of the summer vacation, busy business and illnesses.

### 2.5. Statistical Analysis

We used Stata 14.0 (StataCorp, College Station, TX, USA) to analyze the data and replaced the missing values with the respondents’ mean scores on the items after evaluating the distribution of the 1–5 Likert scale. First, we descriptively analyzed the frequency and percentages of the sample respondents. Second, after reversing negatively worded items to make sure that positive answers presented a high score, the reliability and validity of the questionnaire structure were examined with factor analysis as well as Cronbach alpha. The percent positive ratings (PPRs) were then calculated among the hospital, surgical departments, and other departments. Answers of “agree” or “strongly agree” for the positively worded items indicated positive responses. The PPR refers to the proportion of the cumulative number of positive responses to each item and the total number of respondents. Moreover, the PPRs between the two department groups were compared on every dimension and every item with *t*-tests in an individual respondent level. Third, we used the Pearson X^2^ tests to compare the positive proportion of “patient safety grade” and “number of events reported” between the surgical departments and other departments. Fourth, after estimating the cluster effect of hospitals on the outcome dimensions, the factors affecting the “overall perceptions of safety”, “frequency of events reported”, and outcome variables of “patient safety grade” as well as “the number of events reported” were explored with multiple linear regression, two-level multiple regression, or two-level ordered logistic regression analysis [31] with basic characteristics and 10 other dimensions as independent variables. We tested for between-hospitals variation using the intraclass correlation coefficient (ICC), which represents the proportion of total variability in a given measure that can be attributed to differences between hospitals. It is equal to τ/(τ + σ^2^), where τ refers to between-hospitals variance and σ^2^ refers to within-staff variance [32]. Taking into consideration the potential clustering effect within hospitals, two-level regression models were applied afterward. In traditional regression models, it is assumed that observations are independent of each other, given the predictors. Multilevel models (also called hierarchical models or mixed-effects models) are developed for a specific situation wherein this independent assumption is not met: cluster-level attributes. That means observations are nested within subclassifications, and each subclassification has its own features. This is especially true in the context of Chinese hospitals, where medical staff working in the same hospital tend to have similar experience because they get together in the same team. The mathematical form of the 2-level multiple regression model is as follows:

Standard linear regression:

y_i_ = β_0_ + β_1_X_1i_ + β_2_X_2i_ + … + β_s_X_si_ + e_i_, ei ~ N(0, δ^2^) (1)

Two-level linear regression:

y_ij_ = β_0_ + β_1_X_1ij_ + β_2_X_2ij_ + … + β_s_X_sij_ + u_0j_ + e_ij_ (2)

where i = 1, …, i is the staff level indicator, and j = 1, …, j is the hospital level indicator; y is the outcome dimensions; e_i_ includes the estimated residual error of y and the hospital variation; u_0j_ is the residual error. Similarly, the mathematical form of the 2-level logistic regression model [31] is as follows:

Standard Logistic Regression:

y_ij_ ~ Bern (1, π_ij_) logit (π_ij_) = α + βX_ij_ + Ɛ_ij_ (3)

Two-level logistic model:

y_ij_ ~ Bern (1, π_ij_)
logit (π_ij_) = α_0j_ + βX_ij_ + Ɛ_ij_
α_0j_ = α + μj (4)

where i = 1, …, i is the staff level indicator, j = 1, …, j is the hospital level indicator, y is the result variables, π_ij_ is the probability of every option of result variables for staff i in hospital j, conditional on staff-level risk factors x_ij_. The random effect model expresses that the logit is the sum of hospital-specific intercept α_j_ and effects of staff-specific effects βX_ij_, while the hospital intercept is a random variable with mean α and random variation μj ~ N(0, τ 2).

### 2.6. Ethical Consideration

Formal ethical approval was not needed for this study according to Chinese law. The respondents voluntarily participated in the survey. The consultants in the hospital research department managed the self-administered questionnaire with informed consent, and the returned questionnaires did not show identifying marks. The authors promised the absence of ethical issues, such as data fabrication, double publication, and plagiarism.

## 3. Results

### 3.1. Characteristics of Respondents

A total of 1379 participants completed the HSOPSC questionnaires. Of the 1379 participants, 779 (56.49%) were from surgical units, and 600 (43.51%) were from other units. The overall response rate was 91.93%. Table 1 shows the details on the characteristics of the respondents.

### 3.2. Percent Positive Ratings (PPRs) of Surgical Departments Compared with Those of Other Departments

Factor analysis (KMO = 0.882) showed that the items within one factor did not load onto more than one factor. Initially, the items in this study appeared to form the same factors as the original questionnaire (Table 2) [6]. Additionally, the internal consistency was calculated for every factor, and the Cronbach alphas ranged from 0.31 to 0.87.

Among these 12 safety culture dimensions, the range of PPRs at the hospital level was 32.80–80.29%, that of the surgical departments was between 32.22% and 80.62%, and that of the other departments was from 33.56% to 79.88% (Table 2).

Among the 12 dimensions, those whose PPRs exceeded or equaled 75% were regarded as areas of strength, whereas those whose PPRs were less than or equal to 50% were considered as fields of weakness [33]. Table 2 exhibits that the surgical departments and other units’ strong dimensions included “teamwork within units” as well as “organizational learning and continuous improvement”, and their weak dimensions included “staffing”, “non-punitive response to errors”, as well as “frequency of events reported”.

In the comparison of safety culture between surgical departments and other departments, three dimensions were statistically significant: “non-punitive response to errors” in surgical departments, which was worse than that in other departments (38.68% vs. 40.61%, *p* = 0.001); “supervisor /manager expectations and actions promoting patient safety” in surgical settings, which was weaker than that in others (69.42% vs. 71.63%, *p* = 0.013); and surgical settings’ “hospital management support for patient safety”, which was also lower than that in other settings (61.83% vs. 65.28%, *p* = 0.027). The differences in other safety culture dimensions between two departments had no statistical significance.

Certain patient safety culture items were also considered in the comparison between the two types of departments. The surgical settings showed good performance for the items “Mistakes have led to positive changes here” and “We have enough staff to handle the workload”. The surgical workers agreed strongly with the reversed items “The staff feel like their mistakes are held against them”, “Whenever pressure builds up, my supervisor/manager wants us to work faster, even if it means taking shortcuts”, “Hospital management seems interested in patient safety only after an adverse event happens”, and “The scenario that extremely serious mistakes do not happen around here is only by chance” in comparison with the employees in other units, thereby presenting a weak safety culture in surgical settings. Similarly, the item “The actions of hospital management show that patient safety is a top priority” was also weaker in surgical units than in other units.

### 3.3. Comparison between Surgical Departments and Other Departments Concerning “Patient Safety Grade” and “Number of Events Reported”

The surgical units and other departments had nearly 50% respondents who believed that the “patient safety grade” of their respective departments was “good”. Moreover, the surgical departments and other departments, respectively, had 7.45% and 10.33% respondents who thought that the “patient safety grade” of their departments was “excellent”. In terms of the “number of events reported”, the surgical departments and other departments, respectively, had 49.94% and 58.84% respondents who did not report adverse events in 2015. In addition, more than 25% of the respondents reported 1–2 adverse events in both departments. Few respondents reported more than 10 cases of adverse events. Table 3 shows the details.

Through the X^2^ test, we found a statistical difference (*p* = 0.010 and *p* = 0.025, respectively) in the comparison of “patient safety grade” and “number of events reported” between the surgical units and other departments. Specifically, a small proportion of respondents in surgical departments thought that “patient safety grade” was “excellent/good” compared with that in other departments, but the former had more adverse events reported compared with the latter (Table 3).

### 3.4. Factors Influencing the “Overall Perception of Safety” and “Frequency of Events Reported” in Surgical Departments

In our study, we used the two-level regression with the staff as the first level and the hospital as the second level. The staff characteristics of Table 1 are the variables of the first level, and the second level takes the first 10 HSOPC dimensions as regression (so it excludes the last two dimensions). The two-level zero model (the regression model is empty, i.e., contains no regression variables) of fitting for “overall perception of safety” in surgical departments showed that ICC = 0.031/(0.031 + 0.377) = 0.076 (*p* = 0.025), with the overall perception of safety as the dependent variable. The hospital difference makes a contribution of approximately 7.6% to the change in the scores of the overall perception of safety with statistical significance. Accordingly, the overall perception of safety should be analyzed with a two-level model. With regard to the frequency of events reported, ICC = 0.040/(0.040 + 0.876) = 0.044 (*p* = 0.051), indicating the necessity of multiple linear regression instead of a multiple level model (Table 4).

The results based on the two-level linear regression model verified that only the staff characteristic “Staff position” exerted an influence on the dimension “overall perceptions of safety”. Furthermore, six dimensions (“teamwork within units”, “organizational learning and continuous improvement”, “staffing”, “non-punitive response to errors”, “supervisor/manager expectations and actions promoting patient safety”, and “hospital management support for patient safety”) affected “overall perceptions of safety” with statistical significance. All these six dimensions had a positive correlation with the dimension “overall perceptions of safety” (Table 5).

In terms of “frequency of events reported” based on multiple linear regression, only “years in hospital” was the influencing factor. Only two dimensions, namely, “feedback and communication about errors” and “teamwork across hospital units” exerted an influence on the “frequency of events reported”. When the results of the two dimensions are satisfactory, the “frequency of events reported” is also satisfactory (Table 5).

### 3.5. Factors Affecting “Patient Safety Grade” and “Number of Events Reported” in Surgical Departments

Similar to the previous two-level analysis, the fitting zero model of “patient safety grade” and “number of events reported” in surgical departments indicated that the variation of both outcome variables had statistical significance (*p* < 0.05) in the hospital level (Table 6). Therefore, “patient safety grade” and “number of events reported” should be analyzed with a two-level model with consideration of the clustering effect of hospital medical staff within hospitals. As categorical variables, the models should be carried out as two-level ordered logistic regression models.

Table 7 shows that medical staff in surgical units with hospital work experience of 1–5 years or 16–20 years gave lower patient safety grade than those with less than 1 year of experience in county hospitals. Health workers in surgical settings with 1–5 years, 6–10 years, and 11–15 years of experience gave a higher patient safety grade than those with <1 year of experience in the position. Moreover, the good results of the dimensions “organizational learning and continuous improvement”, “supervisor/manager expectations and actions promoting patient safety”, “feedback and communication about errors”, and “hospital handoffs and transitions” of the respondents equated to a good “patient safety grade”.

With regard to the “number of events reported”, the nurses reported fewer adverse events than the doctors in surgical departments. The staff with tenure of “1–5 years”, “6–10 years”, “11–15 years”, and “>20 years” in the hospital reported more adverse events than the staff with “<1 year” of tenure in surgical settings of county hospitals. The surgical staff working in their unit 80–90 h per week reported fewer adverse events than those working <20 h per week. Respondents who had low scores on “hospital handoff and transition” reported several adverse events (Table 7).

## 4. Discussion

Our research is one of the few studies on the comparison of patient safety culture among different departments in hospitals. In China, this study is the first attempt to explore safety culture issues in surgical departments compared with those in other departments in county hospitals. This study will help test the application of the HSOPSC in China to provide a reference for the safety culture assessment of Chinese county hospitals.

Our study highlighted the advantages of the dimensions “teamwork within units” and “organizational learning and continuous improvement” in surgical settings. Without considering the economic level and hospital effects, we found this result to be different from those of the dimensions with the top two highest PPRs from non-surgical departments of nursing homes [34] and neonatal intensive units [17] although the results are similar to the strength dimensions of surgical units in other regions [26]. Evidently, China can learn from the measures and strategies of the safety culture construction of surgical departments in foreign hospitals to improve the safety culture of surgical departments in county hospitals. Furthermore, the differences of the items showed that the staff in surgical units held better perceptions of “Mistakes have led to positive changes here” and “We have enough staff to handle the workload” than those in other settings. In addition, the proportion of medical staff who reported events was larger in surgical units than in other departments. These findings represent the aspects of county hospitals that should be continuously consolidated.

In this study, certain dimensions were found to have an effect on the outcome dimensions. Six dimensions had a positive influence on the “overall perceptions of safety”, and two dimensions had a good effect on the “frequency of events reported”. Moreover, the dimension “hospital handoffs and transitions” was related to “patient safety grade” and “number of events reported”. The high scores of the former dimension equated to a good “patient safety grade” and “number of events reported”. Therefore, by improving the factors that can affect outcome dimensions and categorical outcomes, such as continuing to strengthen the teamwork within or outside surgical units and organizational learning as well as ensuring good feedback and communication as well as hospital handoffs and transitions, we can indirectly improve the overall safety level of hospitals [26].

Although surgical settings presented their own characteristics and advantages, our study found certain weak dimensions that require improvement. These dimensions included “staffing”, “non-punitive response to errors”, and “frequency of events reported”. In addition, the dimensions “supervisor/manager expectations and actions promoting patient safety” and “hospital management support for patient safety” in surgical settings were lower than those in other settings. Certain items were also much weaker in surgical settings than in others. These items included “The staff feel like their mistakes are held against them”, “Whenever pressure builds up, my supervisor/manager wants us to work faster, even if it means taking shortcuts”, and “Hospital management seems interested in patient safety only after an adverse event happens”, “The scenario that extremely serious mistakes do not happen around is only by chance”, and “The actions of hospital management show that patient safety is a top priority”. In the following paragraphs, we will discuss how to improve the weak dimensions and items.

In the results, “staffing” received the lowest PPRs in surgical settings and others. Accordingly, staffing is the most serious problem concerning patient safety culture. Hellings [35] and Smits [36] also found the same issue. Although the respondents felt good about “whether the surgical units have enough staff to handle the workload”, county hospitals still suffer from the lack of health personnel [37]. Surgical staff face a heavy workload, with nearly 85% of them working more than 40 h per week and more than 25% of them working more than 60 h per week. The large amount of work overload not only leads to mental and physical exhaustion for medical personnel but also results in the emergence of anxiety, depression, and other health problems, all of which may increase the adverse event incidence and are not conducive to patient health [38]. In our study, “staffing” had a positive influence on “overall perceptions of safety”. Consequently, the number of medical staff in Chinese county hospitals should be increased [39]. Meanwhile, it is also important to clear division of labor to ease the workload of medical staff.

The dimension “non-punitive response to errors” showed the second lowest PPRs. Similar to our study, other works found low PPRs for the dimension “non-punitive response to errors” [40,41]. On the one hand, the medical staff may worry that their mistakes will be included in their personal files and that reporting errors may lead to their suffering punishment, although hospitals stipulate no punishment for reported errors. On the other hand, Chinese medical workers may hold a wrong view that the number of adverse events reported shows their poor ability, which may affect their honor and income. This study also corroborated that approximately half of the medical staff in surgical departments and other units had never reported adverse events. The employees’ negligence in reporting adverse events results in the masking of errors, which leads to a vicious cycle of error occurrence. In addition, once the error is not corrected, it may bring serious harm to patients as well as adversely affect the quality and brand of medical service institutions. Evans [42] asserted that a punitive response to errors is a barrier for doctors and nurses to report adverse events. We also elucidated that “non-punitive response to errors” had a positive influence on the “overall perceptions of safety”. Therefore, as “To Err Is Human: Building a Safer Health System” by the IOM pointed out, hospitals should consider abolishing potential rules and regulations on punitive responses for minor medical errors while establishing a mandatory reporting system for those major errors [4]. Only by creating a non-punitive inclusive and open environment can hospitals’ non-punitive culture improve.

The dimensions “supervisor/manager expectations and actions promoting patient safety” and “hospital management support for patient safety”, which were weaker in surgical units than in other units, were also necessary for us to consider. In our study, a small proportion of respondents thought that “The actions of hospital management show that patient safety is a top priority”. They also agreed that “whenever pressure builds up, my supervisor/manager wants us to work faster, even if it means taking shortcuts” and that “hospital management seems interested in patient safety only after an adverse event happens”. Our study is slightly similar to that of Liu [22]. Compared with other units, the surgical units are focused on solving surgical questions instead of considering safety culture building. In the county hospitals of China, supervisors/managers may lack health management basis because they work as surgical doctors at the same time. Supervisors/managers bear a heavy workload that they might have no time to implement management practices, leading to the neglect of patient safety culture. Furthermore, after finishing their work, supervisors/managers may go home or rest, thereby making their communication with employees insufficient. In this case, the staff’s suggestions about safety culture might not be considered by supervisors/managers completely.

Supervisors/managers play an important role in helping their teams establish a culture that offers quality patient care in a safe environment [43]. Furthermore, good leadership is an incentive to improve communication and enhance safety [44]. Among the incentive mechanisms, supervisors/managers’ support and action on employees could prompt the positive work of the latter group. Our study also verified that the support, attitudes, and actions of managers/supervisors toward safety culture in county hospitals did contribute to the overall perception of safety of the respondents and patient safety grade. Consequently, we think that hospital management training could be strengthened to improve the management and consciousness level in surgical units. Similarly, communication with one another may also be advocated to construct a good safety culture in surgical departments [45].

### Limitations

Our study has some limitations. First, the some Cronbach alpha values were low (ranging from 0.31 to 0.87). The reason behind the low Cronbach alpha values could possibly be due to the cultural differences between China and other countries. However, we decided not to delete the dimensions with low internal consistency to compare our results with those of other studies. Second, we compared patient safety culture between surgical units and other units for the results, conclusions, and strategies to be suitable for surgical units in county hospitals and not for other units in other hospitals. In other words, our study has the standard limitation of generalizability. Third, because of the limited sample size, the differences between the two kinds of unit groups are relatively small even if they are statistically significant. Fourth, our present study is similar to our previous study [26], especially on the structure. The previous study [26] has provided a reference for our present study. However, there are still some differences between our research and [26]: (1) The subject and sample size are different. We studied the safety culture of 19 hospitals, while the [26] studied the safety culture of only one tertiary hospital. (2) The method was not completely the same. We used the multilevel statistical method, which was not used by [26]. (3) The content is not all the same. Our study not only compared the 12 dimensions of patient safety culture between surgical and non-surgical departments, but also compared the differences between the 42 items. However, the [26] just compared the 12 dimensions between the two groups. (4) The results were different. The strengths and weaknesses of the patient safety culture in the surgical units of the county hospitals are not all the same as those of the tertiary hospital. Meanwhile, the county hospitals emphasized the management on patient safety culture, which was not mentioned by the former study. (5) Our research strategy proposed according to the research results is not exactly the same as the [26]. Despite these limitations, our methods, results, and strategies may be helpful for those county hospitals willing to assess and improve their patient safety culture.

## 5. Conclusions

Much can be done in the context of county hospitals to improve patient safety in surgical departments. Recruiting workers, using the reporting system as frequently as possible, and building a non-punitive culture were recommended to ensure patient safety. In addition, supervisors should also attach priority to patient safety and play a key role in promoting the culture.

## Figures and Tables

**Table 1:** Characteristics of 1379 respondents based on HSOPSC results.

Characteristics	Kinds	Departments			
		Surgical Departments		Other Departments	
		*n*	%	*n*	%
Staff position	Physician	320	41.08	254	42.33
	Nurse	459	58.92	346	57.67
Years in hospital	<1 year	68	8.73	72	12.00
	1–5 years	296	38.00	233	38.83
	6–10 years	151	19.38	111	18.50
	11–15 years	82	10.53	55	9.17
	16–20 years	81	10.40	66	11.00
	>21 years	101	12.97	63	10.50
Years in department	<1 year	111	14.25	130	21.67
	1–5 years	329	42.23	281	46.83
	6–10 years	129	16.56	91	15.17
	11–15 years	90	11.55	41	6.83
	16–20 years	60	7.70	28	4.67
	>21 years	60	7.70	29	4.83
Years in current profession	<1 year	78	10.01	84	14.00
	1–5 years	379	48.65	304	50.67
	6–10 years	163	20.92	113	18.83
	11–15 years	84	10.78	50	8.33
	16–20 years	42	5.39	27	4.50
	>21 years	33	4.24	22	3.67
Hours worked per week	<20	10	1.28	11	1.83
	20–39	123	15.79	65	10.83
	40–59	447	57.38	343	57.17
	60–79	121	15.53	135	22.50
	80–99	50	6.42	29	4.83
	>100	28	3.59	17	2.83
Average monthly income	<3000 RMB	416	53.40	285	47.50
	3000–5000 RMB	323	41.46	275	45.83
	5000–8000 RMB	37	4.75	36	6.00
	>8000 RMB	3	0.39	4	0.67
Contact with patients	Yes	746	95.76	564	94.00
	No	33	4.24	36	6.00

Notes: HSOPSC is defined as the “Hospital Survey on Patient Safety Culture”. RMB is Renminbi.

**Table 2:** The Percent Positive Ratings of safety culture dimension and items between surgical units and other units.

Kinds	Items	Hospital		Surgical Department		Other Departments		*t*	*p*
		*n*	PPRs (%)	*n*	PPRs (%)	*n*	PPRs (%)		
**Unit level**	**Teamwork within units (Cronbach α = 0.67)**	**4429**	**80.29**	**2512**	**80.62 ^a^**	**1917**	**79.88 ^a^**	**0.205**	**0.838**
	People support one another in this facility.	1196	86.70	679	87.16	517	86.17	0.890	0.373
	When considerable work needs to be done quickly, we work together as a team to get the work done.	1160	84.10	661	84.85	499	83.17	0.912	0.362
	In facility, people treat one another with respect.	1178	85.40	657	84.34	521	86.83	−0.868	0.385
	When one area in this unit gets extremely busy, others help out.	895	64.90	515	66.11	380	63.33	0.427	0.670
	**Organizational learning and continuous improvement (Cronbach α = 0.46)**	**3274**	**79.14**	**1873**	**80.15 ^a^**	**1401**	**77.83 ^a^**	**0.707**	**0.480**
	We are actively doing things to improve patient safety.	1222	88.60	695	89.22	527	87.83	0.823	0.411
	Mistakes have led to positive changes here.	1011	73.30	592	75.99	419	69.83	2.475	0.013 *
	After we make changes to improve patient safety, we evaluate their effectiveness.	1041	75.50	586	75.22	455	75.83	−0.199	0.842
	**Staffing (Cronbach α = 0.31)**	**2026**	**36.73**	**1175**	**37.71 ^b^**	**851**	**35.46 ^b^**	**0.289**	**0.772**
	We have enough staff to handle the workload.	529	38.40	330	42.36	199	33.17	2.257	0.024 *
	Staff in this unit work longer hours than is best for patient care. R	152	11.00	79	10.14	73	12.17	−1.491	0.136
	We use more agency/temporary staff than is best for patient care. R	635	46.00	362	46.47	273	45.50	0.740	0.459
	We work in “crisis mode” trying to do too much, too quickly. R	710	51.50	404	51.86	306	51.00	−0.815	0.415
	**Nonpunitive response to errors (Cronbach α = 0.48)**	**1635**	**39.52**	**904**	**38.68 ^b^**	**731**	**40.61 ^b^**	**−3.303**	0.001 *
	When an event is reported, it feels like the person is being written up, not the problem. R	888	64.40	502	64.44	386	64.33	−1.629	0.103
	Staff worry that mistakes they make are kept in their personnel file. R	321	23.30	185	23.75	136	22.67	−0.846	0.398
	Staff feel like their mistakes are held against them. R	426	30.90	217	27.86	209	34.83	−4.131	<0.001 *
	**Supervisor/manager expectations and actions promoting patient safety (Cronbach α = 0.64)**	**3882**	**70.38**	**2163**	**69.42**	**1719**	**71.63**	**−2.479**	0.013 *
	Supervisor/manager says a good word when he/she sees a job done according to established guidelines.	861	62.40	469	60.21	392	65.33	−1.644	0.101
	My supervisor/manager seriously considers staff suggestions for improving patient safety.	1041	75.50	591	75.87	450	75.00	0.333	0.739
	Whenever pressure builds up, my supervisor/manager wants us to work fast, even if it means taking shortcuts. R	918	66.60	507	65.08	411	68.50	−2.407	0.016 *
	My supervisor/manager overlooks patient safety problems that happen over and over. R	1062	77.00	596	76.51	466	77.67	−1.058	0.290
	**Feedback and communication about errors (Cronbach α = 0.61)**	**2479**	**59.92**	**1425**	**60.98**	**1054**	**58.56**	**1.526**	**0.127**
	We are given feedback about changes put into place on the basis of event reports.	561	40.70	325	41.72	236	39.33	0.915	0.360
	In this unit, we discuss ways to prevent errors from recurring.	1023	74.20	581	74.58	442	73.67	0.121	0.903
	We are informed about errors that happen in this unit.	895	64.90	519	66.62	376	62.67	1.563	0.118
	**Communication openness (Cronbach α = 0.79)**	**2304**	**55.69**	**1307**	**55.93**	**997**	**55.39**	**0.450**	**0.653**
	Staff will freely speak up if they see something that may negatively affect patient care.	995	72.20	569	73.04	426	71.00	0.626	0.531
	Staff feel free to question the decisions or actions of those with substantial authority.	406	29.40	221	28.37	185	30.83	−0.066	0.948
	Staff are afraid to ask questions when something does not seem right. R	903	65.50	517	66.37	386	64.33	0.353	0.724
**Hospital level**	**Hospital handoffs and transitions (Cronbach α = 0.68)**	**3590**	**65.08**	**2038**	**65.40**	**1552**	**64.67**	**−0.088**	**0.930**
	Important patient care information is commonly lost during shift changes. R	1126	81.70	640	82.16	486	81.00	−0.664	0.507
	Problems generally occur in the exchange of information across hospital units. R	902	65.40	505	64.83	397	66.17	−1.512	0.131
	Things “fall between the cracks” when transferring patients from one unit to another. R	621	45.00	358	45.96	263	43.83	0.300	0.764
	Shift changes are problematic for patients in this hospital. R	941	68.20	535	68.68	406	67.67	0.208	0.835
	**Teamwork across hospital units (Cronbach α = 0.62)**	**3604**	**65.34**	**2056**	**65.98**	**1548**	**64.50**	**−0.541**	**0.588**
	Working with staff from other hospital units is generally unpleasant. R	1031	74.80	586	75.22	445	74.17	−0.165	0.869
	Hospital units work well together to provide the best care for patients.	918	66.60	531	68.16	387	64.50	0.177	0.860
	Good cooperation is present among hospital units that need to work together.	769	55.80	431	55.33	338	56.33	0.072	0.943
	Hospital units do not coordinate well with one another. R	886	64.20	508	65.21	378	63.00	0.031	0.975
	**Hospital management support for patient safety (Cronbach α = 0.61)**	**2620**	**63.33**	**1445**	**61.83**	**1175**	**65.28**	**−2.217**	0.027 *
	Hospital management provides a work climate that promotes patient safety.	766	55.50	435	55.84	331	55.17	−0.307	0.759
	The actions of hospital management show that patient safety is a top priority.	1017	73.70	560	71.89	457	76.17	−2.048	0.041 *
	Hospital management is interested in patient safety only after an adverse event happens. R	837	60.70	450	57.77	387	64.50	−3.201	0.001 *
**Outcome dimensions**	**Overall perceptions of safety (Cronbach α = 0.87)**	**3219**	**58.36**	**1835**	**58.89**	**1384**	**57.67**	**0.002**	**0.998**
	The scenario that extremely serious mistakes do not happen around here is only by chance. R	968	70.20	536	68.81	432	72.00	−2.157	0.031 *
	We had patient safety problems in this unit. R	471	34.20	276	35.43	195	32.50	0.521	0.602
	Patient safety is never sacrificed to get considerable work done. R	979	71.00	571	73.30	408	68.00	1.655	0.098
	Our procedures and systems are good at preventing errors from happening.	801	58.10	452	58.02	349	58.17	−0.338	0.735
	**Frequency of events reported (Cronbach α = 0.80)**	**1357**	**32.80**	**753**	**32.22 ^b^**	**604**	**33.56 ^b^**	**0.503**	**0.615**
	When a mistake is made, but is caught and corrected before affecting the patient, how often is this reported?	487	35.30	277	35.56	210	35.00	0.031	0.976
	When a mistake is made, but could harm the patient, how often is this reported?	468	33.90	247	31.71	221	36.83	−1.642	0.101
	When a mistake is made that could harm the patient, but does not, how often is this reported?	402	29.20	229	29.40	173	28.83	1.371	0.171

Notes: “^a^” represents the strength dimensions; “^b^” represents the weak dimensions; * represents the dimensions and items with statistical significance in surgical departments compared with those in other units; “R” represents the negatively worded items.

**Table 3:** Comparison of “patient safety grade” and “number of events reported” between departments.

Outcome Variables	Kinds	Surgical Departments (*n* = 792)	Other Departments (*n* = 1978)	Pearson X^2^
Patient safety grade	Excellent	58 (7.45%)	62 (10.33%)	X^2^ = 11.327 *p* = 0.010
	Good	367 (47.11%)	301 (50.17%)	
	Acceptable	321 (41.20%)	201 (33.50%)	
	Poor/failing	33 (4.24%)	36 (6.00%)	
Number of events reported	No reports	389 (49.94%)	353 (58.83%)	X^2^ = 11.181 *p* = 0.025
	1–2 reports	231 (29.65%)	150 (25.00%)	
	3–5 reports	110 (14.12%)	65 (10.83%)	
	6–10 reports	28 (3.59%)	17 (2.83%)	
	>10 reports	21 (2.70%)	15 (2.50%)	

**Table 4:** Parameter variance estimation in zero model of overall perception of safety and frequency of events reported.

Overall Perceptions of Safety ^a^							Frequency of Events Reported ^b^					
**Fixed Effect Estimation**												
Parameter	Estimate	SE	df	*p*	95% CI		Estimate	SE	df	*p*	95% CI	
Intercept	3.52	0.048	17.92	<0.001	3.419	3.621	3.001	0.06	18.235	<0.001	2.875	3.126
**Random Effect Estimation**												
Parameter	Estimate	SE	Wald Z	*p*	95% CI		Estimate	SE	Wald Z	*p*	95% CI	
Residual	0.377	0.019	19.513	<0.001	0.341	0.416	0.876	0.045	19.527	<0.001	0.792	0.969
Intercept (subject = hospital)	0.031	0.014	2.245	0.025	0.013	0.073	0.04	0.021	1.955	0.051	0.015	0.110

Notes: ^a^ Dependent variable: Overall perceptions of safety; ^b^ Dependent variable: Frequency of events reported.

**Table 5:** Factors influencing “overall perception of safety” and “frequency of events reported” in surgical departments.

Parameter	Overall Perception of Safety ^a^(n = 1379)				Frequency of Events Reported ^b^(*n* = 1379)				Collinearity	
	Estimate	*p*	95% CI		Estimate	*p*	95% CI			
			Lower	Upper			Lower	Upper	Tolerance	VIF ^c^
Intercept	0.082	0.742	−0.409	0.574	1.186	0.006	0.343	2.029		
Staff position	0.089	0.037 *	0.006	0.172	0.012	0.868	−0.133	0.158	0.760	1.316
Years in hospital	−0.027	0.285	−0.076	0.022	0.087	0.048 *	0.001	0.174	0.218	4.594
Years in department	−0.024	0.365	−0.077	0.028	−0.047	0.319	−0.140	0.046	0.220	4.546
Years in current profession	0.006	0.778	−0.038	0.051	−0.013	0.745	−0.091	0.065	0.438	2.284
Hours worked per week	0.027	0.201	−0.014	0.068	0.053	0.148	−0.019	0.124	0.848	1.180
Average monthly income	0.010	0.761	−0.057	0.077	0.083	0.149	−0.030	0.195	0.843	1.187
Contact with patients	0.136	0.144	−0.047	0.319	0.109	0.503	−0.211	0.430	0.939	1.065
Teamwork within units	0.169	<0.001 *	0.089	0.249	0.016	0.826	−0.123	0.154	0.545	1.834
Organizational learning and continuous improvement	0.147	<0.001 *	0.067	0.226	0.117	0.100	−0.023	0.257	0.582	1.717
Staffing	0.144	<0.001 *	0.067	0.220	−0.046	0.492	−0.179	0.086	0.859	1.164
Nonpunitive response to error	0.095	0.001 *	0.041	0.149	−0.006	0.903	−0.101	0.089	0.754	1.327
Supervisor/manager expectations and actions promoting patient safety	0.179	<0.001 *	0.100	0.258	0.043	0.541	−0.095	0.181	0.510	1.960
Feedback and communication about errors	0.027	0.415	−0.038	0.091	0.469	0.000 *	0.356	0.581	0.528	1.893
Communication openness	−0.029	0.423	−0.099	0.041	−0.104	0.098	−0.227	0.019	0.573	1.746
Hospital handoffs and transitions	0.009	0.789	−0.058	0.077	0.022	0.718	−0.096	0.139	0.575	1.739
Teamwork across hospital units	0.066	0.125	−0.019	0.151	0.267	0.000 *	0.118	0.415	0.438	2.282
Hospital management support for patient safety	0.067	0.037 *	0.004	0.131	0.087	0.117	−0.022	0.196	0.502	1.991

Notes: * represents the dimensions and items with statistical significance in surgical departments; ^a^ Two-level multiple linear regression model: the first level comprises medical staff in surgical units, and the second level comprises county hospitals; ^b^ Multiple linear regression model; ^c^ VIF: Variance inflation factor. All VIFs < 5, showing no collinearity among the independent variables.

**Table 6:** Parameter variance estimation in zero model of patient safety grade and number of events reported.

	Estimate	SE	Z	*p* > |Z|	(95% Conf. Interval)	
**Patient Safety Grade**						
Excellent	−2.614	0.177	−14.800	<0.001	−2.960	−2.268
Good	0.152	0.132	1.150	0.250	−1107.000	0.410
Acceptable	3.168	0.211	15.010	<0.001	2.754	3.582
Poor/failing	5.099	0.463	11.020	<0.001	4.192	6.007
Hospital Var(_cons)	0.197	0.109			0.066	0.583
LR test vs. ologit model: chibar2(01) = 11.88, Prob ≥ chibar2 = 0.0003						
**Number of Events Reported**						
No events	0.105	0.160	0.650	0.513	−0.209	0.418
1–2 Events	1.583	0.171	9.260	<0.001	1.248	1.918
3–5 Events	3.001	0.212	14.140	<0.001	2.585	3.417
6–10 Events	3.854	0.270	14.270	<0.001	3.325	4.383
>10 Events	5.081	0.435	11.670	<0.001	4.228	5.935
Hospital Var(_cons)	0.336	0.150			0.140	0.806
LR test vs. ologit model: chibar2(01) = 48.51, Prob ≥ chibar2 = 0.0001						

Notes: LR test is conservative and provided only for reference; Pro ≥ chibar2 equals to *p* value.

**Table 7:** Factors influencing “patient safety grade” and “number of events reported” in surgical departments based on two-level ordered logistic regression.

Threshold	PSG Reference Category: PSG = Excellent					NER Reference Category: NER = “>10 Events”			
	Estimate	*p*	95% CI			Estimate	*p*	95% CI	
PSG = good	−10.433	<0.001	−12.648	−8.218	NER = no events	−0.265	0.801	−2.325	1.795
PSG = acceptable	−7.185	<0.001	−9.323	−5.048	NER = 1–2 events	1.363	0.195	−0.697	3.423
PSG = poor/failing	−3.672	0.001	−5.774	−1.571	NER = 3–5 events	2.880	0.006	0.806	4.954
					NER = 6–10 events	3.760	<0.001	1.658	5.861
**Staff Position**									
Physician	0 ^a^					0 ^a^			
Nurse	0.071	0.761	−0.281	0.423		−0.409	0.024 *	−0.763	−0.054
**Years in Hospital**									
<1 year	0 ^a^					0 ^a^			
1–5 years	−0.140	0.761	−1.039	0.760		1.031	0.030 *	0.101	1.960
6–10 years	−0.601	0.258	−1.642	0.441		1.586	0.003 *	0.525	2.648
11–15 years	−0.321	0.601	−1.525	0.883		2.033	0.001 *	0.801	3.264
16–20 years	0.011	0.986	−1.218	1.240		2.320	<0.001 *	1.066	3.573
>21 years	−0.282	0.656	−1.526	0.961		2.691	<0.001 *	1.450	3.931
**Years in Department**									
<1 year	0 ^a^					0 ^a^			
1–5 years	−0.702	0.047 *	−1.393	−0.010		−0.106	0.762	−0.796	0.583
6–10 years	−0.597	0.199	−1.508	0.314		−0.825	0.074	−1.729	0.080
11–15 years	−0.056	0.918	−1.120	1.008		−0.639	0.235	−1.694	0.416
16–20 years	−1.161	0.046 *	−2.300	−0.021		−1.068	0.063	−2.194	0.058
>21 years	−0.202	0.740	−1.392	0.989		−0.912	0.122	−2.067	0.243
**Years in Current Profession**									
<1 year	0 ^a^					0 ^a^			
1–5 years	1.181	0.004 *	0.372	1.989		−0.189	0.645	−0.992	0.614
6–10 years	1.307	0.006 *	0.378	2.235		0.317	0.495	−0.595	1.230
11–15 years	1.122	0.029 *	0.112	2.133		−0.057	0.910	−1.046	0.931
16–20 years	1.006	0.068	−0.074	2.087		−0.234	0.671	−1.312	0.844
>21 years	1.043	0.096	−0.184	2.270		−0.545	0.378	−1.756	0.667
**Hours Worked per Week**									
<20 h	0 ^a^					0 ^a^			
20–39 h	0.043	0.949	−1.276	1.363		−0.759	0.231	−2.000	0.482
40–59 h	0.156	0.811	−1.124	1.436		−0.934	0.128	−2.136	0.268
60–79 h	0.147	0.828	−1.176	1.469		−0.827	0.191	−2.065	0.412
80–99 h	0.136	0.851	−1.289	1.562		−1.812	0.009 *	−3.173	−0.451
>100 h	−0.184	0.809	−1.676	1.308		−0.910	0.206	−2.320	0.500
**Average Monthly Income**									
<3000 RMB	0 ^a^					0 ^a^			
3000–5000 RMB	0.118	0.507	−0.231	0.467		0.173	0.318	−0.166	0.512
5000–8000 RMB	0.322	0.414	−0.451	1.095		−0.061	0.866	−0.770	0.647
>8000 RMB	−1.382	0.224	−3.609	0.845		−21.124	0.999	−41.25	−10.237
**Contact with Patients**									
Yes	0 ^a^					0 ^a^			
No	0.176	0.668	−0.627	0.978		−0.014	0.972	−0.818	0.790
Teamwork within units	−0.081	0.630	−0.412	0.249		−0.195	0.241	−0.520	0.131
Organizational learning and continuous improvement	0.489	0.004 *	0.152	0.826		0.022	0.896	−0.305	0.349
Staffing	0.104	0.522	−0.214	0.422		0.079	0.620	−0.234	0.392
Nonpunitive response to error	−0.173	0.134	−0.400	0.053		0.092	0.420	−0.132	0.317
Supervisor/manager expectations and actions promoting patient safety	0.368	0.031 *	0.034	0.703		0.147	0.373	−0.177	0.472
Feedback and communication about errors	0.347	0.012 *	0.076	0.618		0.240	0.080	−0.028	0.509
Communication openness	0.083	0.577	−0.209	0.375		0.095	0.516	−0.191	0.381
Hospital handoffs and transitions	0.477	0.002 *	0.171	0.782		0.418	0.003 *	0.142	0.695
Teamwork across hospital units	−0.175	0.343	−0.537	0.187		−0.173	0.328	−0.519	0.174
Hospital management support for patient safety	−0.168	0.220	−0.435	0.100		0.079	0.557	−0.184	0.341

Notes: Join function: Logit. ^a^ This parameter is set to 0 because it is redundant. CI: confidence interval, PSG: patient safety grade, NER: number of events reported. * represents the dimensions and items with statistical significance in surgical departments. PSG: Patient safety grade. NER: Number of events reported.

## Abbreviations

CI	confidence interval
HSOPSC	Hospital Survey on Patient Safety Culture
PPRs	percent positive ratings
AHRQ	Agency of Healthcare Research and Quality
RMB	Ren Min Bin
PSG	patient safety grade
NER	number of events reported
